# Tumors as complex organs: are cancers manageable through the modification of their microenvironment?

**DOI:** 10.1186/1753-6561-7-S2-K16

**Published:** 2013-04-04

**Authors:** Roger Chammas

**Affiliations:** 1 Center for Translational Cancer Research, Instituto do Câncer do Estado de São Paulo and Faculdade de Medicina da Universidade de São Paulo, São Paulo, Brazil

## 

Cancers behave as organoids, composed not only by genetically altered tumor cells, but also by a variety of non-tumoral cells, such as fibroblasts, macrophages and endothelial cells among others. Cellular interactions within this organoid dictate the rate of tumor progression, and therefore the accumulation of heterogeneous genotypes which characterize the tumor mass at the moment of diagnosis. Evidence for both homotypic and heterotypic cooperation between cells within the tumor microenvironment exist. We will show evidence for fluctuations in the expression of a specific tumor marker, namely galectin-3.

Galectin-3 is an animal lectin that is found in diverse cellular compartments, depending on the cellular context and functional state of cells. Normal cells tend to accumulate galectin-3 in the nucleus. Upon malignant transformation, galectin-3 may concentrate in the cytoplasm and can be secreted to the extracellular matrix, where it acts as a matricellular protein, interfering with integrin function among other cell surface glycoproteins. We had shown a focal accumulation of galectin-3 in glioblastomas. Galectin-3 expression was associated to a very specific tumor microenvironment, the pseudopalisades- areas of viable tumor cells surrounding necrotic areas that are commonly found in advanced stages of glioblastomas. Galectin-3 expression in hypoxic and nutrient deprived cells is associated the activation of HIF-1a and it was inhibitable by NF-kB antagonists. In glioblastoma cells, galectin-3 expression was associated with its translocation to mitochondria, suggesting its involvement in mitochondrial homeostasis. Inhibition of galectin-3 expression led to increased cell death, indicating that galectin-3 acts as a prosurvival factor in very specific tumor microenvironments associated with glioblastoma progression. The protumoral role of galectin-3 was also observed in murine melanomas, engrafted in both wild type and galectin-3-deficient mice. Absence of galectin-3 from both tumoral and stromal compartments was associated with attenuated tumor growth, as compared to an experimental condition where both tumor and stromal cells expressed galectin-3. Intriguingly, de novo expression of galectin-3 was frequently observed in tumors derived from cells that did not express galectin-3 when these cells were engrafted in galectin-3 deficient mice. This observation prompted us to investigate whether galectin-3 expressing cells would favor tumor growth when admixed with galectin-3 negative cells. Indeed, when cells are admixed, tumors grew much more efficiently than those engraftments of either cell alone, indicating cooperation between galectin-3 expressing cells and galectin-3 negative cells. Further experiments indicated that galectin-3 expression is related with the induction of a proangiogenic tumor environment. Thus, targeting galectin-3 expression in selected tumors may prove to be an effective strategy to control tumor growth.

Angiogenesis and vasculature function are critically altered in cancers, which are usually ill-perfused. Evidence for activation of both angiotensin and bradykinin-dependent pathways within the tumor microenvironment of melanomas will be discussed. Blockage of vasoactive peptide-dependent pathways may control tumor angiogenesis and therefore may control tumor growth. Indeed, epidemiological studies are now indicating that individuals receiving specific blockers of angiotensin receptors may be relatively protected from the development of tumors. In a clinical setting, however, antiangiogenic drugs would be preferably used in combination with chemotherapeutic drugs. Vasculature function, however is also critical for the proper delivery of chemotherapeutic drugs to tumors, specific therapeutic windows for the application of antiangiogenic drugs need to be identified. This notion will be further discussed. Altogether, the examples above call for a necessary understanding of tumor physiology, which seems to be under appreciated in the design of experimental/clinical trials.

## Competing interests

There are no competing interests in this presentation.

